# Osteosarcoma: A Comprehensive Morphological and Molecular Review with Prognostic Implications

**DOI:** 10.3390/biology14101407

**Published:** 2025-10-13

**Authors:** Alessandro El Motassime, Raffaele Vitiello, Rocco Maria Comodo, Giacomo Capece, Guido Bocchino, Maria Beatrice Bocchi, Giulio Maccauro, Cesare Meschini

**Affiliations:** 1 Department of Orthopedics and Geriatric Sciences, Catholic University of the Sacred Heart, 00168 Rome, Italy; alessandro.elmotassime01@icatt.it (A.E.M.); raffaele.vitiello@policlinicogemelli.it (R.V.); roccocomodo96@gmail.com (R.M.C.); giacomo.capece01@icatt.it (G.C.); guido.bocchino01@icatt.it (G.B.); mariabeatrice.bocchi01@icatt.it (M.B.B.); giulio.maccauro@policlinicogemelli.it (G.M.); 2 Department of Orthopedics and Rheumatological Sciences, Fondazione Policlinico Universitario A. Gemelli IRCCS, 00168 Rome, Italy; 3 Department of Orthopaedics, Mater Olbia Hospital, Strada Statale 125 Orientale Sarda, 07026 Olbia, Italy; 4 U.O.C. Orthopedics and Traumatology, Ospedale dei Pellegrini, 80134 Naples, Italy

**Keywords:** osteosarcoma, bone tumor, tumor microenvironment, immune biomarkers, molecular pathways

## Abstract

Osteosarcoma (OS) is the most common primary malignant bone tumor, predominantly affecting adolescents and young adults. Despite advances in surgery and multi-agent chemotherapy, survival rates for metastatic or recurrent OS remain poor, highlighting the need for novel prognostic and therapeutic strategies. This review integrates histopathologic, molecular, and immune perspectives to provide a comprehensive understanding of OS biology in the context of precision medicine. We discuss classic morphologic and radiographic features alongside recent insights into the tumor microenvironment, including the role of tumor-infiltrating lymphocytes, tumor-associated macrophages, and immune checkpoint expression. Emerging molecular markers, such as gene expression–based immune risk signatures, circulating tumor DNA, and gasdermin D overexpression, are evaluated for their prognostic and therapeutic relevance. Key dysregulated pathways, including WNT/β-catenin and JAK/STAT, are examined in relation to metastasis, chemoresistance, and immune evasion, with emphasis on current targeted approaches under development. By bridging histopathology, immunogenomics, and translational research, this work outlines how integrated biomarker assessment can refine patient stratification and guide the implementation of individualized treatment strategies in OS.

## Simple Summary

This review provides a comprehensive overview of the morphological, radiological, histopathological, immunohistochemical, and molecular features of osteosarcoma, highlighting their prognostic implications. High-grade osteosarcoma encompasses conventional subtypes—osteoblastic, chondroblastic, and fibroblastic—as well as rarer surface and secondary variants with distinctive clinical and pathological profiles; classic radiological features and novel radiogenomic approaches are also highlighted for their potential to refine risk stratification. We also summarize emerging prognostic biomarkers, such as immune infiltration patterns, circulating tumor DNA, and novel molecular markers including gasdermin D, which hold promise for refining patient prognosis and guiding personalized therapy. While survival rates for localized disease have improved with multimodal treatment, outcomes remain poor for metastatic or relapsed osteosarcoma. By integrating traditional pathology with advances in genomics, immunology, and imaging, this review provides an updated resource for clinicians, researchers, and students, emphasizing the importance of precision medicine in improving future osteosarcoma management.

## 1. Introduction

Osteosarcoma (OS) is a malignant mesenchymal neoplasm and is typically defined by its ability to produce osteoid or immature bone by the tumor cells [1,2]. The world incidence of OS is low, at approximately 2–4 per million persons per year, and is equivalent to some 800–900 new cases per year in the United States [3,4]. Although fairly rare in all cancers, OS warrants significant clinical interest as the most common primary bone sarcoma, with a predisposition for ages in children and adolescents. Clinically and histologically refractory, OS has a broad spectrum that can vary from indolent and localized to extremely aggressive and metastatic disease [5].

The epidemiology of OS presents bimodal age distribution: one large peak in adolescents during times of rapid skeletal growth and one secondary, smaller peak in older adults, typically associated with disorders of the bone such as Paget’s disease or prior irradiation. Risk factors include raised adolescent height, raised birth weight, radiotherapy (prior radiation therapy is causally linked with the development of high-grade OS subtypes, with prominent dose–response, typical latency of 8–15 years, and a predisposition to axial and craniofacial sites [6,7]), and genetic predisposition syndromes—autosomal dominant (e.g., Li-Fraumeni syndrome, hereditary retinoblastoma, Diamond-Blackfan anemia) and autosomal recessive (e.g., Rothmund-Thomson syndrome, RAPADILINO syndrome, Werner syndrome, and Bloom syndrome). Candidate gene and genome-wide association studies suggest a multifactorial genetic etiology with contributions from rare and common variants [8,9].

Comprehension of the interaction between tumor biology, the microenvironment of the bone, and host factors is crucial to decode the clinical heterogeneity of OS. While the major morphological characteristics have been recognized for over a century, recent advances in molecular pathology, immunology, and imaging have substantially enhanced both diagnostic precision and therapeutic strategies. Notably, whole transcriptome analyses have provided deeper insights into OS biology, highlighting molecular fingerprints that distinguish tumor tissue from normal bone and clarifying mechanisms of transcriptional deregulation [10,11]. However, prognosis in the case of advanced or metastatic disease still remains poor, highlighting the necessity of further investigation into the pathogenesis of OS.

This review summarizes the key features of OS, with particular focus on macroscopic and histopathological characteristics, immunohistochemical profiles, and prognostic markers. The aim is to provide a comprehensive and up-to-date resource for clinicians, pathologists, and researchers.

## 2. Macroscopic Features

Osteosarcoma presents with heterogeneous gross appearances reflecting its biological heterogeneity [12,13,14]. High-grade conventional OS, which includes osteoblastic, chondroblastic, and fibroblastic variants, is the most frequent subtype in adolescents, typically arising in metaphysis of long bones, commonly in distal femur, proximal tibia, or proximal humerus. The tumors are ill-defined, infiltrative, and may replace the affected bone and spread into adjacent soft tissues through a cortical break. Cut surfaces are gritty due to the mineralized matrix and consist of a mixture of solid, hemorrhagic, necrotic, and cystic elements. Cartilaginous zones, necrosis, and palpable ossification can arise, and periosteal elevation can lead to the characteristic Codman triangle [15].

The remaining OS subtypes, defined as non-conventional OS, possess distinct macroscopic features. Parosteal OS is typically low-grade, and it is a lobulated, hard, exophytic mass with possible cartilaginous caps [16]. Periosteal OS presents as having a broad base and thickened cortex with chondroid elements. High-grade surface OS, while uncommon, is similar to conventional high-grade OS in gross appearance. Those occurring in older individuals or patients with predisposing conditions (e.g., Paget’s disease or previous radiation) are larger, more destructive, and occur in unusual locations like the pelvis, jaw, or axial skeleton with large soft tissue components and central necrosis. These are characteristics that hold across high-grade subtypes [17].

## 3. Radiological Features

Radiologic evaluation is crucial for detection, characterization, and staging of OS in all the subtypes [18,19,20,21]. Plain X-rays may reveal the classic “sunburst” periosteal reaction and Codman triangle. MRI is essential to assess marrow involvement, soft tissue involvement, and skip lesions, and CT is used to assess cortical lysis and pulmonary metastasis [22]. PET scans are used for staging and assessing response to therapy [23,24].

Emerging approaches—radiogenomics and deep learning—enable the extraction of radiomic features that have the potential to non-invasively forecast treatment response and histologic subtypes. PET/CT-based parameters such as metabolic tumor volume and total lesion glycolysis relate to event-free survival and histologic response, whereas MRI signal patterns are correlated with immune phenotypes, bridging imaging characteristics with underlying tumor biology [25].

Radiological features of OS differ according to histologic subtype, reflecting underlying matrix composition and tumor biology [26,27,28]. Osteoblastic OS typically appears predominantly sclerotic, with dense, cloud-like bone formation and aggressive periosteal reaction, emphasizing its osteoid-rich nature [18,26]. In contrast, chondroblastic OS often shows mixed areas of chondroid matrix—characterized by rings-and-arcs calcifications—interspersed with osteoid, maintaining a similarly aggressive periosteal response [28]. Fibroblastic OS tends to be more lytic, with less conspicuous matrix mineralization, reflecting its collagen-dominant composition [18]. Telangiectatic OS presents as a purely lytic, expansile lesion, frequently demonstrating fluid-fluid levels on MRI due to blood-filled cystic spaces, which can mimic aneurysmal bone cyst [18,26].

Surface variants display distinctive imaging patterns: parosteal OS, typically low-grade, manifests as a heavily ossified, lobulated mass with a broad cortical attachment and often a radiolucent cleavage plane separating tumor from cortex [18]. Periosteal OS, an intermediate-grade lesion, shows a soft tissue mass with chondroid matrix and a prominent perpendicular (“sunburst”) periosteal reaction, though ossification is less than in parosteal or high-grade surface types [18,26,29]. High-grade surface OS appears as a broad-based, surface-attached soft tissue mass with moderate to dense, immature mineralization; periosteal reaction is less pronounced than in periosteal OS, and while medullary involvement is uncommon at presentation, it may develop over time [18].

Finally, secondary OS, arising in settings such as prior radiation or Paget disease, often exhibits aggressive radiographic features, including extensive bone destruction, poorly defined margins, and large soft tissue components. Matrix mineralization is typically reduced, and multifocal lesions are more frequently observed, particularly in Paget-associated cases [18]. Overall, these radiologic differences provide important clues to subtype identification and guide surgical planning, while correlating with underlying histopathology.

## 4. Histopathology

The hallmark of OS is malignant osteoid or immature bone production. Tumor cells in conventional high-grade OS are pleomorphic, show hyperchromatic nuclei, and increased mitotic activity, with invasion into both cortex and medullary cavity. Osteoid is a lace-like unmineralized matrix alongside tumor cells. According to the latest World Health Organization (WHO) classification, OS subtypes are defined as follows [30,31] in Table 1.

Conventional OS comprises several variants distinguished by their predominant extracellular matrix. The osteoblastic subtype is characterized by abundant deposition of osteoid and bone matrix, whereas the chondroblastic form demonstrates a rich cartilaginous component. In the fibroblastic subtype, spindle-shaped cells produce dense collagen with only limited osteoid, while the telangiectatic variant appears as cystic spaces filled with blood, separated by malignant septa. Less common forms, such as the giant cell-rich and small cell variants, are rare and of uncertain prognostic significance [14,18,30,32].

Beyond conventional subtypes, low-grade central OS may mimic fibrous dysplasia both radiologically and histologically, but its permeative growth pattern and potential for dedifferentiation distinguish it from benign conditions. Surface OSs represent another group: parosteal OS is a low-grade tumor composed of mature bone with minimal atypia, periosteal OS shows intermediate-grade features with a prominent chondroid matrix and moderate atypia, while the high-grade surface subtype is histologically indistinguishable from conventional high-grade OS [14,18].

Finally, secondary OSs develop in the context of predisposing conditions, most commonly Paget’s disease of bone or previous radiation exposure, and are generally associated with more aggressive clinical behavior and poorer prognosis [18].

## 5. Differential Diagnosis

The differential diagnosis of OS includes a variety of benign, malignant, and infectious conditions that can mimic its clinical and radiographic features, such as localized pain, bone destruction, and occasionally soft-tissue masses. Accurate differentiation requires integration of clinical history, imaging, and histopathology, often supported by molecular and immunohistochemical studies.

Among malignant tumors, Ewing sarcoma is the second most common primary bone malignancy in children and young adults, presenting with pain, swelling, and sometimes fever. Radiographs typically reveal diaphyseal, lytic lesions with a permeative “moth-eaten” pattern and onion-skin periosteal reaction, while histology shows sheets of small round blue cells with MIC2/CD99 positivity and EWSR1 translocation, without osteoid formation [33,34,35]. In adults, chondrosarcoma arises de novo or from pre-existing cartilage lesions, presenting with pain and lytic lesions containing chondroid matrix; histology confirms malignant cartilage rather than osteoid [35,36,37].

Infectious lesions such as osteomyelitis or Brodie abscess can mimic OS, often with systemic symptoms like fever. Imaging may show lytic areas with sclerosis or sequestra, and histology reveals necrotic bone with inflammatory infiltrates, but no malignant cells [38,39]. Metastatic bone disease is another important consideration in adults, with lytic, blastic, or mixed lesions reflecting the primary tumor and lacking osteoid, frequently causing pain or pathologic fracture [34,40].

Benign bone-forming tumors, including osteoid osteoma and osteoblastoma, may appear aggressive. Osteoid osteoma causes nocturnal pain relieved by NSAIDs and shows a small nidus with reactive sclerosis, while osteoblastoma is larger and expansile; both display woven bone with benign osteoblasts without malignant features [36,40,41]. Aneurysmal bone cysts may mimic telangiectatic OS, with fluid–fluid levels on MRI, but biopsy reveals blood-filled cystic spaces with fibrous septa containing giant cells and no osteoid [32,34]. Giant cell tumors arise in epiphyses of skeletally mature adults, are locally aggressive, and histologically show osteoclast-like giant cells amid mononuclear stromal cells without osteoid [34,42,43].

Rare mimics include primary bone lymphoma and acute leukemia. Primary lymphoma usually presents as a solitary permeative lesion with minimal cortical destruction and a soft-tissue mass in older patients; biopsy shows sheets of atypical lymphoid cells expressing CD45. Acute leukemia may diffusely affect the skeleton, presenting with bone pain, systemic symptoms, and radiographic osteopenia or lytic lesions, necessitating hematologic testing for definitive diagnosis [43,44].

The key distinguishing feature of OS lies in the concordance of clinical, radiological, and histopathological findings. Clinically, it typically presents in adolescents with progressive pain and swelling. Radiologically, it demonstrates mixed lytic and sclerotic lesions, aggressive periosteal reactions such as the sunburst pattern or Codman’s triangle, and often an associated soft-tissue mass. Most decisively, histopathology reveals malignant cells producing osteoid matrix, a pathognomonic hallmark. In contrast, Ewing sarcoma is identified by its small round cell morphology and characteristic molecular translocations, chondrosarcoma by malignant cartilage formation, and osteomyelitis by inflammatory rather than neoplastic changes. Osteoid osteoma, osteoblastoma, aneurysmal bone cyst, and giant cell tumor are benign lesions with distinctive histologic patterns but without malignant osteoid. Metastases and leukemia are recognized based on clinical context and histology.

In conclusion, while several lesions can mimic OS clinically and radiographically, malignant osteoid formation remains the definitive diagnostic characteristic. Diagnoses necessitate a multidisciplinary correlation of patient age, clinical presentation, radiographic pattern, laboratory results, and ultimate histopathological and molecular analysis.

Table 2 provides an overview of the key clinical, radiologic, and histopathologic features of common primary and secondary bone lesions, facilitating differential diagnosis.

## 6. Immunohistochemistry

Immunohistochemistry (IHC) represents a cornerstone in the diagnostic workup of OS, both to confirm the osteoblastic lineage and to exclude histological mimics. Among the most reliable markers, SATB2 is widely expressed across all subtypes and strongly supports osteoblastic differentiation, while osteocalcin and osteonectin indicate active osteoid production [45,46]. These markers help consolidate the diagnosis, particularly in poorly differentiated cases.

In specific subtypes, such as low-grade central and parosteal OS, MDM2 and CDK4 overexpression provide additional diagnostic clues, facilitating their distinction from benign fibro-osseous lesions [47,48]. Conversely, negative markers such as S100 and SOX10 (neural/chondrogenic lineage), or CD31 and CD45 (vascular/hematopoietic origin), are useful in ruling out histological mimics [49].

Beyond diagnosis, several immunohistochemical markers carry potential prognostic relevance. For instance, ZFP36 downregulation has been linked to worse survival, while GBP2 expression appears to contribute to immune activation and tumor suppression [22]. Immune checkpoint molecules such as PD-L1 are currently under investigation as potential predictors of response to immunotherapy, although their definitive role in OS remains to be clarified [50].

Table 3 outlines the immunohistochemical markers relevant for the diagnostic and prognostic assessment of OS.

## 7. Molecular Pathology and Tumor Microenvironment

### 7.1. Genetic Alterations

Osteosarcoma has extensive chromosomal instability with no pathognomonic translocations [46], as shown in Table 4. Integrative sequencing studies, combining whole exome and transcriptome approaches, have confirmed the heterogeneity of OS and revealed patient-specific mutational landscapes that can be mapped against gene expression data [51]. Among the most frequent genetic alterations, TP53 loss is consistently observed across all subtypes and represents a hallmark of tumor suppressor inactivation [45]. Mutations of RB1, a key regulator of the cell cycle, are also recurrent and have been associated with poor prognosis [48]. MDM2 amplification, instead, is typical of low-grade OSs such as parosteal and central forms, reflecting specific pathway dysregulation. In addition to these canonical changes, aberrations in signaling cascades including MYC, WNT/β-catenin, JAK/STAT, and TGF-β contribute to tumor progression and aggressiveness [47]. Furthermore, ALPL, the gene encoding tissue-nonspecific alkaline phosphatase, is highly expressed in OS cells, highlighting its essential role in bone mineralization and its relevance in tumor biology [49,50].

Pharmacogenetic studies have identified gene signatures (e.g., ABCC1, ABCG2, GSTP1) of drug resistance [52]. Gene modules of immunoblasts (e.g., PRKD2, PRF1, SP140) modulate survival [22]. miR-21, miR-221, and miR-29b are involved in tumor aggressiveness [53].

ALPL expression is a hallmark of OS and is used as a diagnostic and prognostic biomarker. At the cellular level, higher ALPL expression is inversely related to tumor aggressiveness; OS cells with high ALPL activity show reduced invasiveness and metastatic potential, likely due to upregulation of cell adhesion genes (e.g., CDH13, CAV1). Conversely, loss or low expression of ALPL is associated with a more malignant phenotype [4,52].

ALPL (specifically the ALPL-1 isoform) is a promising immunotherapeutic target: CAR-T cells directed against ALPL-1 have demonstrated efficacy in preclinical OS models, indicating clinical significance for targeted therapy [54].

Table 4 summarizes the spectrum of genetic alterations identified in OS, with potential implications for prognosis and therapeutic strategies.

### 7.2. Tumor Microenvironment

The host microenvironment—consisting of tumor-infiltrating lymphocytes (TILs), macrophages, and stromal components—is significant in OS development modulation, immunity, and response to therapy. Heavy TIL infiltration is generally correlated with good prognosis and is common in low-risk tumors that are positive for immune checkpoint molecules such as PD-L1 [55,56,57].

Tumor-associated macrophages (TAMs) and neutrophils regulate OS progression conditionally based on local cytokine signatures and immune activation states, supporting or suppressing growth depending on the context [58,59,60].

Recent single-cell RNA sequencing studies have revealed novel immune cell subpopulations and cell-to-cell communication between cancer and host cells. Moreover, subsets of OS cells exhibit activation of the unfolded protein response (UPR), with ATF6α identified as a key contributor of tumor malignancy. Genetic or pharmacological inhibition of ATF6α has been shown to interfere with tumor survival and is a potentially exploitable therapeutic target [61,62]. Additionally, differential network regulation between OS and normal bone identified by transcriptome-wide studies further emphasizes the interplay between tumor cells and their microenvironment [63].

## 8. Survivorship

Survivorship in OS also heavily relies on histological subtype. For conventional high-grade central OS, the 5-year overall survival is approximately 60–65% in large series [64,65,66].

Among conventional subtypes, the osteoblastic form shows a 5-year overall survival of around 62%, while the chondroblastic variant reaches about 60%, with outcomes generally comparable to those of the fibroblastic subtype [5,13,67]. No significant differences in overall or cancer-specific survival have been consistently demonstrated between chondroblastic and fibroblastic cases [5,13,44]. In contrast, the fibroblastic subtype is associated with more favorable outcomes, with a 5-year overall survival approaching 83%, significantly higher than other conventional forms [13,67,68]. The telangiectatic variant also carries a relatively good prognosis, with survival rates of 75–80%, exceeding those of osteoblastic and chondroblastic tumors [69].

Prognosis among surface OSs reflects their histological grade. Parosteal OS, a low-grade surface tumor, is associated with excellent long-term outcomes, with 10-year overall survival rates exceeding 90%, often achieved with surgical resection alone [70]. Periosteal OS, of intermediate grade, shows intermediate behavior, with survival usually above 80% at 10 years [70]. By contrast, high-grade surface OS behaves aggressively, with 10-year overall survival below 50%, underscoring its poor prognosis despite similarities in histology to conventional high-grade central OS [70].

In summary, fibroblastic and telangiectatic subtypes have the optimal prognosis of the high-grade central OSs, and parosteal and periosteal (surface) subtypes have the optimal prognosis overall. High-grade surface OS has a distinctly worse prognosis than other surface subtypes [61,71]. These variations in survival are reproduced reliably in large multi-institutional series and meta-analyses [72].

The survivorship of most common OS subtypes is presented in Table 5.

## 9. Prognostic Biological Factors

### 9.1. Established Prognostic Factors

The prognosis of OS is decided by a complex combination of clinical, anatomical, histopathological, biochemical, molecular, and imaging parameters. Presenting metastatic disease remains the most powerful unfavorable prognostic factor, with 5-year survival rates of less than 30%. Tumor characteristics, including size and axial location, are associated with poor outcomes, whereas smaller, appendicular tumors generally offer an improved prognosis. Age at presentation is a survival determinant, with both older patients and very young patients doing poorly.

Following neoadjuvant chemotherapy, an over 90% tumor necrosis is a good prognostic indicator, and residual tumor cells normally found in retraction clefts or in fibrotic tissues carry an increased risk of recurrence [4]. Surgical margins are also significant; marginal or infiltrating resections significantly enhance the risk of local recurrence and mortality [73]. Elevated serum alkaline phosphatase (ALP) and lactate dehydrogenase (LDH) are correlated with tumor burden and bad prognosis [74,75]. At a molecular level, gene expression–based risk models (e.g., GBP2, PLEKHO2, MPP1, VSIG4) have been shown to have prognostic significance, but high tumor purity correlates with more aggressive disease [4]. Emergent imaging also adds precision to risk stratification: PET/CT-derived radiomic features, including metabolic tumor volume and total lesion glycolysis, are prognostic for histologic response and event-free survival, and MRI patterns are associated with immune phenotypes, emphasizing associations between imaging characteristics and tumor biology [76,77]. Integration of these multidimensional variables gives an integrative system of personalized prognostication and treatment planning in OS [69]. Table 6 reports the various prognostic factors along with the strength of evidence.

### 9.2. Controversial/Novel Factors

In addition to well-established prognostic factors, several controversial or emerging variables have been investigated in OS. Pathologic fracture, although consistently associated with an increased risk of local recurrence, does not appear to uniformly translate into poorer overall survival. The prognostic value of histologic subtype also remains debated: while most conventional variants show comparable outcomes, dedifferentiated and high-grade surface OSs are generally linked to less favorable survival.

Among novel biomarkers, circulating tumor DNA (ctDNA) has attracted significant interest as a tool for disease monitoring and early detection of relapse, although its integration into clinical practice is still pending standardization [80]. Similarly, gene expression–based signatures are being explored for their predictive potential: a recently proposed 5-gene model (ERCC4, GPX4, EPS8, TERT, STAT5A) has demonstrated the ability to discriminate between high- and low-risk groups [81]. From an immunological perspective, T-cell exhaustion signatures have emerged as relevant classifiers, allowing stratification of patients into immune-high and immune-low subgroups, with significant implications for prognosis and potential therapeutic decision-making.

### 9.3. Emerging Molecular and Immune Markers

New prognostic and therapeutic OS biomarkers have been outlined in recent research, a reflection of the evolving interplay between tumor biology and host immune response. Prognostically relevant gene expression–based models built on TILs markers have been described, with “high-risk” molecular profiles predictive of compromised immune infiltration and poor outcomes. Aberrant signaling pathways, such as WNT/β-catenin and JAK/STAT, are associated with increased metastatic ability, chemoresistance, and immune evasion, and represent candidates for therapeutic modulation [67,79].

Gasdermin D. (GSDMD) is a new promising biomarker: overexpression in the tumor is linked with aggressive clinical behavior and poor pathological features, and its expression has been recently identified as an independent survival prognosis factor [78]. Gasdermin D. expression in OS has been implicated in immunogenic cell death and modulation of the tumor immune microenvironment. Recent molecular profiling demonstrates that GSDMD, a key effector of pyroptosis, is variably expressed across OS samples and is associated with increased immune cell infiltration and a more immunogenic microenvironment, which may correlate with improved prognosis and responsiveness to immunotherapy [24]. Coupling GSDMD assessment with other immune and molecular markers may increase patient stratification and allow to identify the candidates for targeted or immunotherapy.

Additional novel markers are T-cell exhaustion profiles, which categorize patients into immune-high or immune-low subgroups with varied prognostic value, and ctDNA, which is of interest for non-invasive monitoring of disease but needs standardization and clinical verification [13,44]. Single-cell RNA sequencing studies reveal substantial infiltration of exhausted T cells in OS, with higher exhaustion scores associated with poor prognosis, reduced immune cell infiltration, and lower responsiveness to immunotherapy [13,44]. There is evidence that recurrent and metastatic lesions, which are more common in secondary OS, display increased immune infiltration and terminally exhausted CD8+ T cells [68].

Overall, the integration of molecular, immunologic, and functional biomarkers like GSDMD, gene expression models, immune infiltration profiles, and changes in signaling pathways holds the promise to further improve prognostication and guide individualized therapeutic regimens in OS.

## 10. Considerations and Perspectives

The management of OS remains a major challenge in musculoskeletal oncology, primarily due to its aggressive biology, clinical heterogeneity, and lack of pathognomonic genetic alterations. As highlighted in this review, variability at the histologic, radiologic, molecular, and immunologic levels complicates both diagnosis and treatment planning. Traditional prognostic factors—such as tumor size, metastatic status at diagnosis, and histologic response to chemotherapy—remain cornerstones of clinical risk assessment. However, their predictive value is not always consistent. For example, although tumor necrosis >90% after neoadjuvant chemotherapy has long been considered a robust favorable prognostic indicator, recent studies have shown conflicting results, suggesting that its predictive power may be context-dependent and influenced by the complexity of tumor–host interactions [4,78]. Similarly, while histologic subtype is often considered in risk stratification, its prognostic relevance remains controversial, with some series reporting no significant survival differences across conventional subtypes.

Beyond these established factors, new molecular and immunological insights are beginning to reshape our understanding of disease biology. Biomarkers derived from the tumor microenvironment—such as T-cell exhaustion gene signatures, tumor-infiltrating lymphocyte (TIL) profiles, and tumor purity scores—have shown potential in stratifying patients into prognostically distinct groups. Nevertheless, these findings still require validation in large multicenter cohorts before they can be integrated into clinical practice [22,82]. The same applies to circulating tumor DNA (ctDNA), which is emerging as a promising non-invasive biomarker for disease monitoring and early detection of relapse. While several studies have highlighted its utility in tracking minimal residual disease, technical and interpretative challenges continue to limit its routine use. Still, liquid biopsy approaches are likely to transform surveillance protocols in the near future by enabling earlier interventions compared to conventional imaging.

A particularly novel molecular marker under investigation is gasdermin D (GSDMD). Lin et al. demonstrated in 2020 that GSDMD is significantly overexpressed in OS tissue and correlates with aggressive clinical features, including higher-grade histology and increased metastatic potential [83]. Importantly, GSDMD expression was identified as an independent adverse prognostic factor, with strong association to reduced overall survival in OS patients [22,82]. This adds a new layer of evidence suggesting that apoptotic and pyroptotic signaling pathways may influence OS progression and could represent novel therapeutic targets.

In parallel, integrative transcriptomic and immune profiling studies have revealed gene-expression models capable of predicting treatment response, including signatures associated with mifamurtide responsiveness and classification of patients into immune-high versus immune-low risk groups. These findings suggest the possibility of tailoring therapy to an individual’s tumor immunophenotype [24,84]. However, clinical translation has been hampered by lack of assay standardization, inconsistent definitions of immune phenotypes, and limited representation of OS patients in large immunotherapy trials.

Another promising field is the application of next-generation sequencing (NGS) for diagnosis and treatment planning. Comprehensive genomic profiling allows for the identification of rare actionable mutations and provides insight into tumor heterogeneity. Nevertheless, its widespread use is constrained by high costs, limited access, and challenges in data interpretation and integration into daily workflows. Moving forward, international collaboration will be essential to harmonize molecular diagnostic pipelines, establish OS-specific bioinformatic platforms, and ensure reproducibility of results across centers [85,86].

In summary, significant advances have been made in characterizing the biological complexity of OS, but the translation of these insights into clinical benefit remains incomplete. Bridging this gap will require multidisciplinary strategies that integrate histopathology, radiomics, immunogenomic

## 11. Conclusions

Osteosarcoma represents a biologically complex cancer with deep histologic and molecular heterogeneity. While conventional diagnostic modalities remain cornerstones, advances in genomics, immunology, and radiomics are enhancing our ability to risk-stratify patients and identify novel prognostic biomarkers.

Despite these advancements, however, outcomes for high-grade and metastatic disease remain bleak. The incorporation of molecular profiling, immune characterization, and pharmacogenomics into the clinic is paramount to the design of personalized, risk-adapted therapy.

Additional translational research and validation of new biomarkers are needed to move towards precision medicine and improve prognosis in OSs, incorporating pharmacogenomics into a unified prognostic framework. Such an approach holds the potential not only to refine risk stratification but also to guide personalized treatment strategies, including the rational selection of patients for novel targeted and immune-based therapies.

## Figures and Tables

**Table 1:** OS subtypes’ characteristics.

Subtype	Prevalence	Grade	Matrix Type	Typical Age	Site	Prognosis
Osteoblastic	68–70%	High	Osteoid	Adolescents	Long bone metaphysis	Intermediate–poor
Chondroblastic	10–19%	High	Cartilage	Adolescents	Long bones	Similar to conventional
Fibroblastic	6–9%	High	Collagen/osteoid	Adolescents	Long bones	Similar to conventional
Telangiectatic	0.4–6%	High	Blood-filled cysts	Adolescents	Long bones	Similar to conventional
Parosteal	~2%	Low	Mature bone	Adults	Posterior distal femur	Good
Periosteal	~0.6%	Intermediate	Cartilaginous	Young adults	Tibia	Intermediate
High-grade surface	<1%	High	Osteoid	Young adults	Diaphysis	Poor
Secondary	5–7%	High	Osteoblastic	Old adults	Axial skeleton	Poor

**Table 2:** Conditions in the differential diagnosis of OS and their characteristics.

Pathology	Clinical Features	Radiologic Features	Histopathologic Features
OS	Pain, swelling, often in adolescents; may have pathologic fracture	Mixed lytic/sclerotic lesion, aggressive periosteal reaction (sunburst, Codman triangle), soft-tissue mass	Malignant cells producing osteoid matrix
Ewing sarcoma	Pain, swelling, fever, systemic symptoms; children/adolescents	Diaphyseal lytic lesion, “moth-eaten” appearance, onion-skin periosteal reaction, soft-tissue mass	Sheets of small round blue cells, MIC2/CD99+, EWSR1 translocation
Chondrosarcoma	Pain, swelling; adults	Lytic lesion with chondroid matrix calcification, endosteal scalloping	Malignant cartilage cells, chondroid matrix
Osteomyelitis (incl. Brodie abscess)	Pain, fever, systemic symptoms; any age	Lytic lesion, possible sequestrum/involucrum, periosteal reaction, Brodie abscess: well-defined lytic lesion with sclerotic rim	Necrotic bone, inflammatory infiltrate, no malignant cells
Bone metastases	Pain, history of primary malignancy; adults	Lytic or blastic lesions, multiple sites, less aggressive periosteal reaction	Tumor cells from primary site (e.g., carcinoma)
Osteoid osteoma	Nocturnal pain relieved by NSAIDs; young adults	Small (<2 cm) radiolucent nidus with surrounding sclerosis	Well-circumscribed nidus of woven bone, benign osteoblasts
Osteoblastoma	Pain, not typically nocturnal; young adults	Larger (>2 cm) lytic lesion, less sclerosis than osteoid osteoma	Similar to osteoid osteoma but larger, benign osteoblasts
Aneurysmal bone cyst	Pain, swelling; children/young adults	Expansile, lytic lesion, fluid-fluid levels on MRI	Blood-filled cystic spaces, septa with giant cells
Giant cell tumor of bone	Pain, swelling; skeletally mature adults	Eccentric, lytic lesion abutting articular surface	Numerous osteoclast-like giant cells, mononuclear stromal cells
Primary bone lymphoma	Pain, swelling, possible systemic symptoms; adults	Lytic lesion, soft-tissue mass, vertebral involvement	Sheets of atypical lymphoid cells, CD45+
Acute leukemia (skeletal involvement)	Bone pain, systemic symptoms (anemia, bleeding, fever)	Diffuse osteopenia, metaphyseal bands, lytic lesions	Leukemic infiltration of marrow, blasts

**Table 3:** Immunohistochemistry markers and their features.

Marker	Expression in OS	Diagnostic Use	Prognostic Value
SATB2	Positive	Confirms osteoblastic lineage	None established
Osteocalcin/ Osteonectin	Positive	Confirms bone production	None
MDM2/CDK4	Positive in low-grade OS	Differentiate from benign lesions	Unclear
S100/SOX10	Negative	Excludes chondroid/neural tumors	—
CD31/CD45	Negative	Excludes vascular or hematopoietic tumors	—
ZFP36	Downregulated	—	Poor prognosis when low
GBP2	Variable	—	Immune activation/tumor suppression

**Table 4:** Genetic alterations and their features.

Gene/Pathway	Role in OS	Diagnostic/Therapeutic Potential
TP53	Tumor suppressor loss	Diagnostic (Li-Fraumeni); limited therapy
RB1	Cell cycle control	Genetic risk; poor prognosis
MDM2 Amplification	Seen in low-grade OS	Diagnostic in parosteal OS
WNT/β-catenin	Aberrant signaling	Research phase
miRNAs (e.g., miR-21, miR-29b)	Expression dysregulated	Prognostic
TERT	Associated with proliferation	Prognostic; limited therapy
ALPL	Expression dysregulated	Prognostic; therapy

**Table 5:** OS subtypes and their 5 and 10 years survival percentages.

Subtype	5-Year Survival	10-Year Survival
Parosteal	90–97%	97%
Periosteal	80–90%	~80%
High-grade surface	50–60%	40–50%
Conventional (overall)	60–71%	59–60%
Osteoblastic	62%	Not specified
Chondroblastic	60%	Not specified
Fibroblastic	83%	Not specified
Telangiectatic	75–80%	Not specified
Unspecified conventional	67–71%	Not specified

**Table 6:** Prognostic factors for OS and their features.

Factor	Prognostic Implication	Strength of Evidence	References
Metastasis at diagnosis	Poor (<30% 5-yr survival)	Strong	Meltzer, NEJM, 2021 [4]; Bielack, JCOOJASCO, 2023 [78]
Tumor size/location	Larger or axial = worse	Strong	Bielack, JCOOJASCO, 2023 [78]
Age (older/very young)	Poor	Moderate	Bielack, JCOOJASCO, 2023 [78]; Papakonstantinou, Cancer Epidemiol, 2024 [79]
Chemotherapy response	>90% necrosis = good; residual viable cells = poor	Strong	Meltzer, NEJM, 2021 [4]; Bielack, JCOOJASCO, 2023 [78]
Surgical margins	Incomplete resection = poor	Strong	He, IJSLE, 2016 [73]
Serum ALP/LDH	High = worse	Moderate	Bacci, Cancer, 2006 [74]; Sever, JCM, 2025 [75]
Gene expression models	Risk stratification (e.g., GBP2, PLEKHO2)	Emerging	Meltzer, NEJM, 2021 [4]
Tumor purity	High = worse (↑aggressiveness)	Emerging (bioinformatic)	Meltzer, NEJM, 2021 [4]
Imaging biomarkers	PET/CT and MRI predict response	Emerging	Huang, Front Oncol, 2025 [76]; Miwa PloS One, 2013 [77]

## Data Availability

No new data were created.

## Abbreviations

The following abbreviations are used in this manuscript:

OS	Osteosarcoma
GSDMD	Gasdermin D
ctDNA	Circulating tumor DNA
LDH	Lactate dehydrogenase
ALP	Alkaline phosphatase
TAM	Tumor associated macrophages
TIL	Tumor infiltrating lymphocytes
UPR	Unfolded protein response
IHC	Immunohistochemistry
WHO	World health organization
